# High-Resolution Ultrasound-Guided Facial Adipose Tissue Restructuring: A Precise Approach to Rejuvenation in a Clinical Case

**DOI:** 10.7759/cureus.104848

**Published:** 2026-03-08

**Authors:** Gladys Velazco, Angela Herrera, Jeremmy Helen Gutierrez Alvarez

**Affiliations:** 1 Anatomical Sciences, Centro Latinoamericano de Entrenamiento Médico e Investigación (CLEMI), Bogota, COL; 2 Molecular Biology, Universidad de los Andes, Merida, VEN; 3 Medicine, Universidad de Carabobo, Valencia, VEN

**Keywords:** adipose tissue, facial adipose tissue, facial adipostructure, facial ultrasound, facial ultrasound pattern, skin

## Abstract

Quantifying the variations in thickness and echogenicity that occur in different facial layers due to the senescence process is challenging. This study aimed to verify the changes in the ultrasound pattern after three sessions of facial adipostructuring using high-frequency ultrasound. This was a prospective, longitudinal, observational study of a 42-year-old female patient, selected based on specific clinical criteria for facial rejuvenation treatment. Ultrasound images were acquired using a 16-20 MHz multifrequency high-resolution ultrasound scanner (Clarius L20, Clarius Mobile Health, British Columbia, Canada) at two facial sites: the superficial adipose compartment of the malar and nasolabial regions, before and after three sessions of facial adipostructuring. Among the most relevant ultrasound findings after applying the adipostructuring sessions, the nasolabial panniculus showed a sonographic modification in the arrangement pattern, specifically in the subcutaneous cellular tissue. This included an improvement in the connective tissue fibers that support all the adipose cells. The malar panniculus showed a slight thickening, increasing from 1.5 to 1.7 millimeters in thickness. In conclusion, the synergy between high-resolution ultrasound and facial adipostructuring establishes a new standard in aesthetic medicine and facial harmonization, where precision, safety, and objective evaluation replace the empirical approach. It not only acts as an intraoperative map but also as a scientific validation tool that allows for the quantification and statistical demonstration of the benefits of facial rejuvenation, elevating adipostructuring to an intervention rigorously based on evidence.

## Introduction

Facial aging is a multifactorial process involving changes in bone, ligaments, muscles, and skin, and, most importantly, alterations in the distribution and biomechanics of subcutaneous fat compartments. In recent years, facial adipose tissue has gone from being considered a simple volumetric reservoir to being recognized as a dynamic actor in orofacial harmony, which has driven the development of minimally invasive techniques aimed at its structural modulation [1]. In this context, facial adipostructure emerges, defined as a technique aimed at the paniculopathic reorganization of facial fat compartments based on their structure, physiology, and biomechanics, without tissue removal under any circumstances. This therapeutic approach seeks to restore the three-dimensional architecture of the face, optimize anti-gravity forces, and recover harmonious facial proportions through selective intervention in the superficial and deep adipose panniculus. Various clinical reports have shown that adipostructuring can significantly improve the signs of facial aging with favorable safety profiles, positioning itself as an alternative or complement to other rejuvenation strategies [2].

Ultrasound imaging of the skin and soft tissues has established itself as a very useful tool in aesthetic medicine, as it allows real-time evaluation of facial stratigraphic anatomy and location [3]. Ultrasound parameters such as subepidermal low-echogenic band (SLEB) [4] can be used to evaluate the aging process and its improvement. The objective of this article is to present a clinical case of ultrasound-guided facial adipose tissue restructuring, describing the anatomical-functional reasoning, the methodology used, and the clinical and ultrasound findings, in order to propose an integrated approach that contributes to optimizing the safety and efficacy of this technique.

## Case presentation

In this case, a prospective, longitudinal, observational study was conducted focusing on a 42-year-old female patient. The clinical evaluation took place in Valencia, Carabobo State, Venezuela. Her reason for consultation was "dissatisfaction with visible signs on her face." Following a detailed facial clinical analysis, she was diagnosed with deflation, loss of facial contour, dark circles under the eyes, frontal and glabellar wrinkles, and lipomatosis, making her a perfect candidate for the study sample.

Before beginning the facial adipostructuring sequence, the patient was interviewed verbally to ensure that she met the following inclusion criteria using a questionnaire with the following questions: a) not having undergone any orofacial harmonization treatment in the last 12 months; b) not having any skin lesions; c) not being pregnant or breastfeeding; d) no medical conditions or medical treatment; e) no immunological problems; f) no history of cancer; and g) no synthetic materials in the facial or cervical region, such as metal implants or non-absorbable fillers.

**Figure 1:**
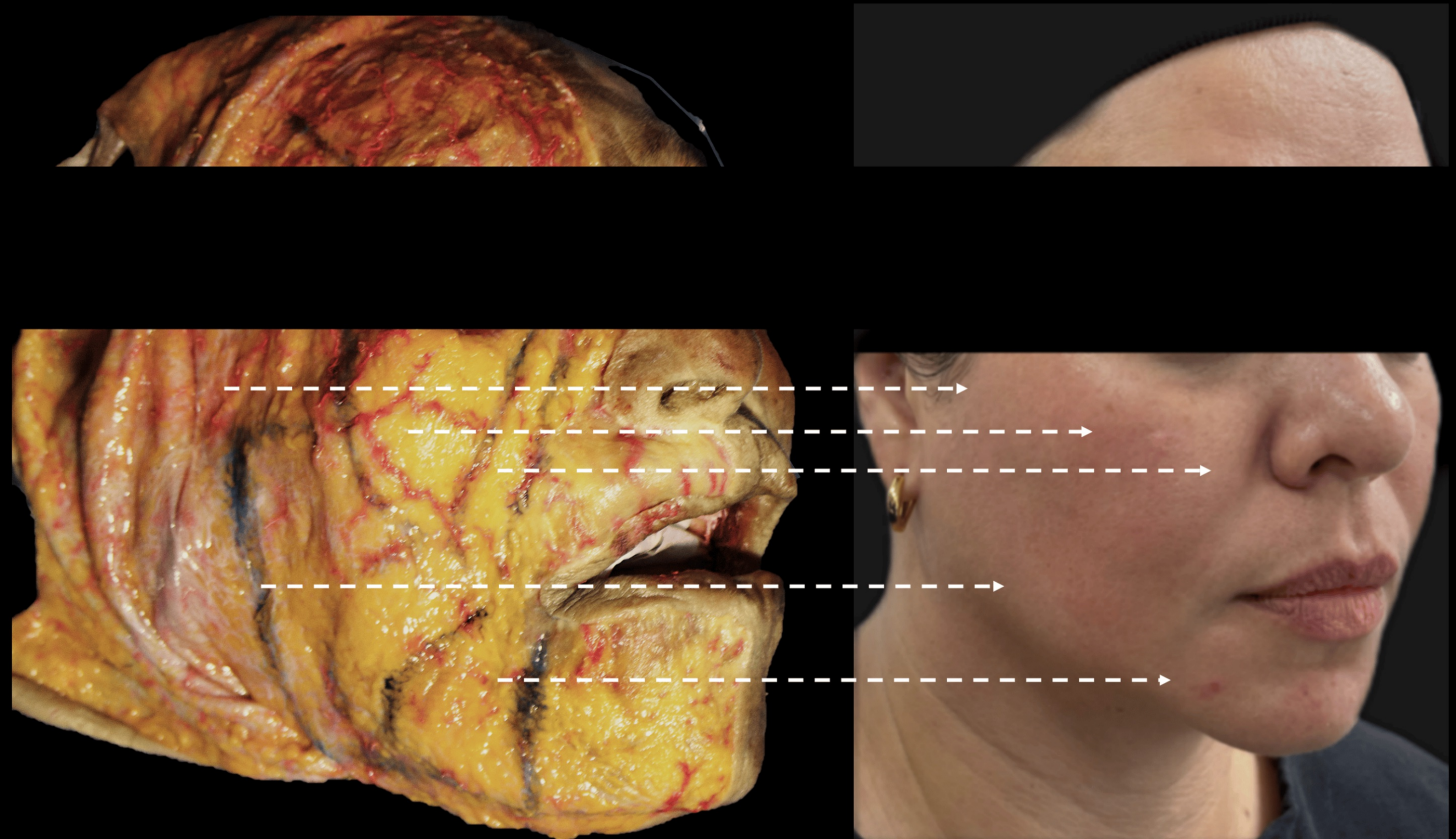
An anatomical comparison using a superficial dissection of the areas treated with the facial adipostructure technique. The location on the patient's face is shown analogically in a photograph using white dots. Latin American Center for Medical Training and Research (CLEMI), Bogota, November 2025.

Following the clinical interview, each step of the facial adipostructuring protocol was explained, along with the benefits and aftercare instructions. The patient signed an informed consent form, acknowledging all the information gathered and confirming their understanding of the procedure, potential risks, and expected results. To conclude this stage, photographic documentation was performed, including frontal, 45°, and profile views. Ultrasound images were acquired using a high-resolution, multifrequency 16-20 MHz ultrasound scanner (Clarius L20, Clarius Mobile Health, British Columbia, Canada) at two facial points: the superficial adipose compartment of the malar region and the nasolabial fold. Both procedures were repeated after the third facial adipostructuring session to visually and sonographically evaluate the morphofunctional changes and the echogenicity pattern. Figure 1 shows an anatomical comparison using a superficial dissection of the areas treated with the facial adipostructure technique, based on a dissection performed at the Latin American Center for Medical Training and Research (CLEMI) in Bogota, November 2025. Figure 1 shows analogically which areas of fatty tissue were treated in comparison with a fresh cadaver dissection.

Materials and technique applied

**Figure 2:**
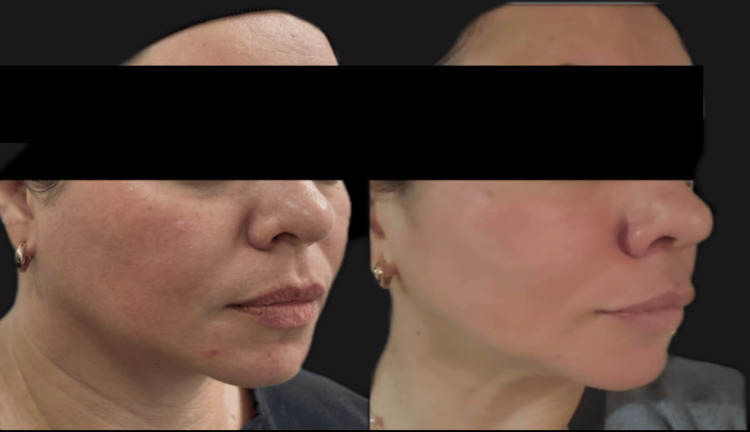
Photographic record of a 42-year-old female patient before and after three sessions of facial adipostructuring.

The patient underwent three sessions at 15-day intervals. The technique was performed using 22G and 25G x 50 mm cannulas, according to the established diagnosis for superficial adipose tissue, and 27G x 50 mm cannulas for ligaments and interseptal spaces, in 1 ml syringes loaded with the senolytic active ingredients (FaceStructure Kit by Mioface Harmony, Cúcuta, Colombia). The treatment was carried out in the following steps: 1. Asepsis with 70% alcohol solution. 2. Marking (face paint). 3. Access perforation with a needle corresponding to the 22G or 25G cannula. The cannula was then inserted, working the superficial adipose compartments with mechanical stimulation through its three manual stages, with three to five movements per vector marking each adipose tissue, finishing with the retro-injection of the selected senolytic agents. 4. Once the adipose compartments were completed, the interseptal spaces were accessed by opening them with the needle corresponding to the 27G cannula. The cannula was then inserted without movement, depositing the corresponding senolytic active ingredient via retro-injection. 5. Postoperative instructions. Figure 2 shows a comparison of before and after the three sessions of adipose restructuring.

**Figure 3:**
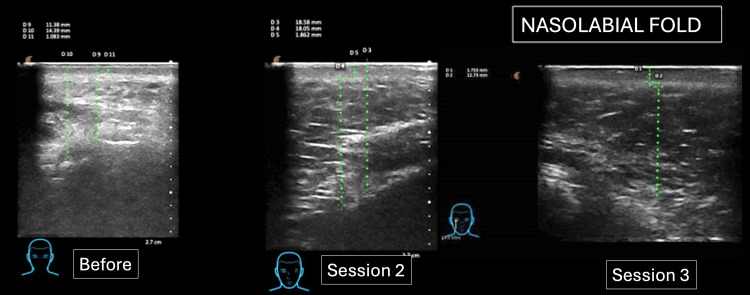
Ultrasound evaluation of the nasolabial fold of a 42-year-old patient, showing the SLEB in the upper area along the dotted green line and its progression over the three sessions. SLEB: subepidermal low-echogenic band.

Among the most relevant ultrasound findings, a change in the arrangement pattern was observed in the treated area, specifically in the connective tissue fibers that support all the adipose cells of the nasolabial panniculus. There were also subtle changes in echogenicity and echostructure (Figure 3).

**Figure 4:**
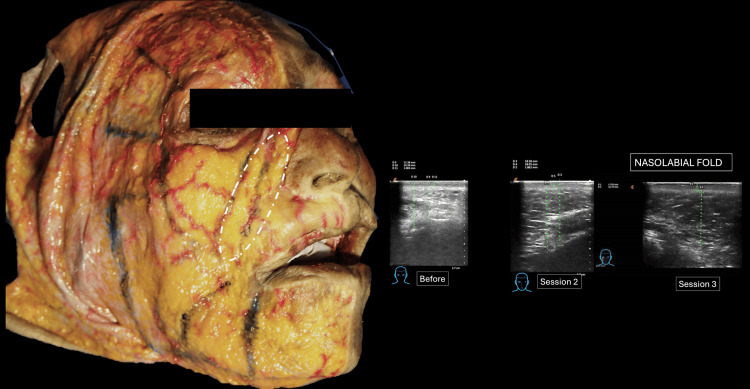
Comparative analysis of the ultrasound assessment with the anatomical component that locates the treated area. The oval shape is defined as the limits of the nasolabial panniculus. Latin American Center for Medical Training and Research (CLEMI), Bogota, November 2025.

Its precise anatomical location is schematically represented through the dissection performed (Figure 4).

**Figure 5:**
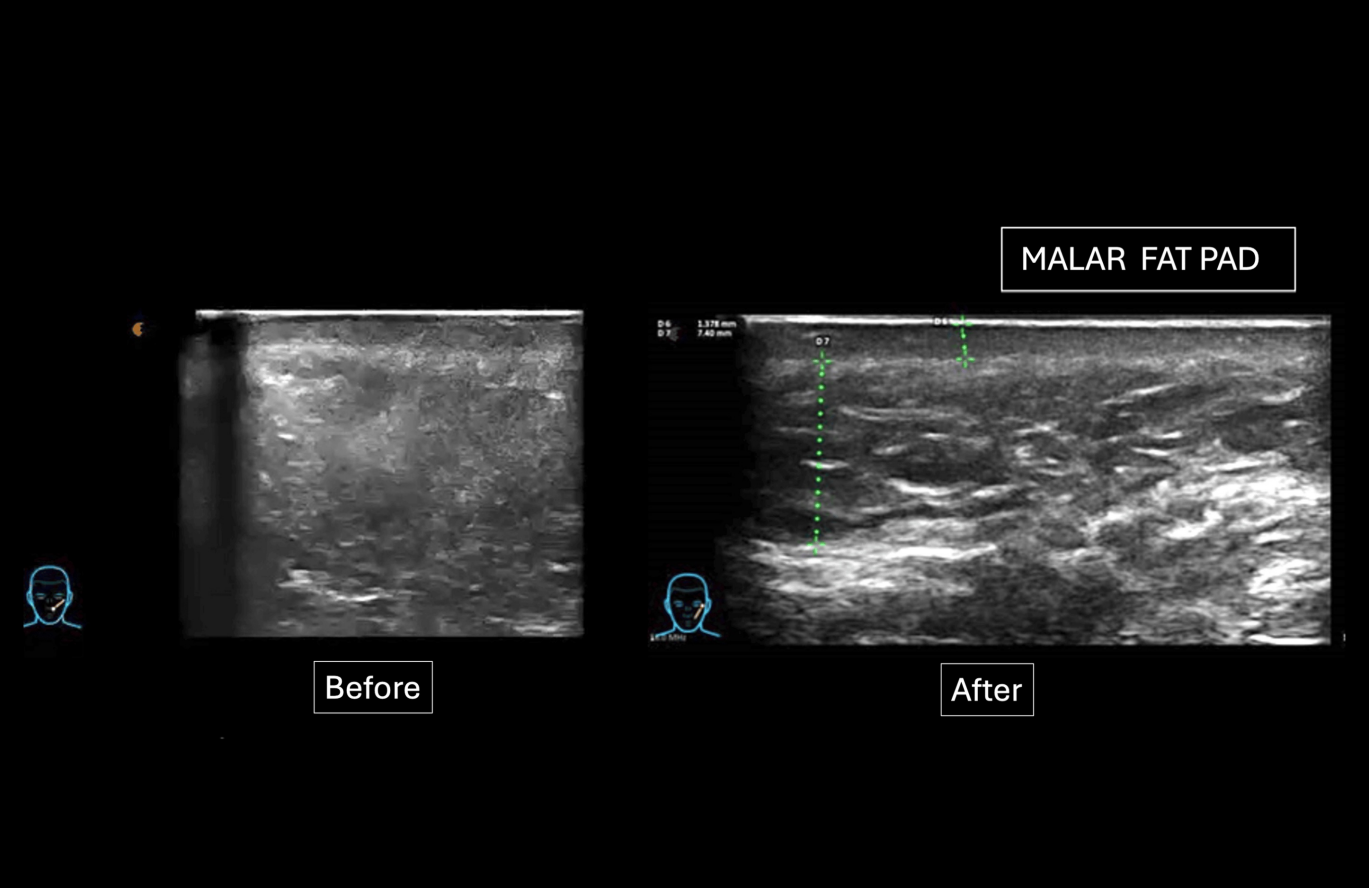
Evaluation of the malar area after adipose tissue transfer. A before-and-after comparison shows improved fat projection and an increase in the SLEB. SLEB: subepidermal low-echogenic band.

Meanwhile, in the malar panniculus, compared to the treated area, a slight thickening was observed, increasing from 1.5 mm to 1.7 mm in thickness, which is associated with repositioning (Figure 5).

**Figure 6:**
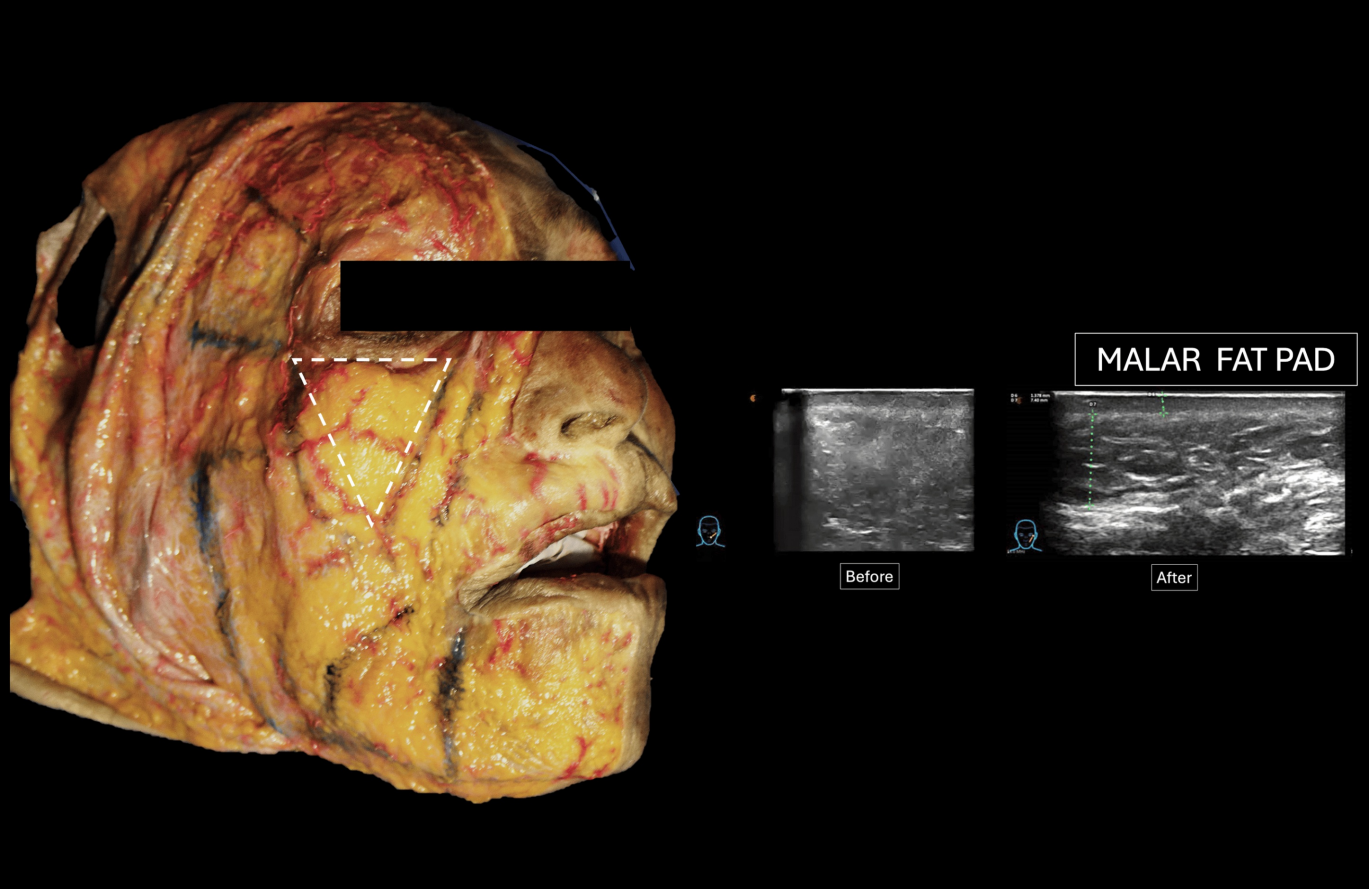
Comparative analysis of the anatomical location of the treated tissue in the malar area. The triangle outlined in the anatomical component describes the limits of the malar panniculus. Latin American Center for Medical Training and Research (CLEMI), Bogota, November 2025.

Its detailed anatomical location can be seen in the schematic representation of the dissection (Figure 6).

## Discussion

After three sessions of facial adipostructure with the FaceStructure Kit, researchers specializing in facial harmonization, with experience in adipostructure and facial ultrasound, demonstrated, through clinical observation and photographic documentation (Figure 2), significant morphofunctional changes in the three facial thirds. The technique's effectiveness in restructuring and repositioning pathognomonic clinical signs was particularly noteworthy. These improvements included a reduction in volumetric increase, a decrease in the nasolabial folds, repositioning of the malar skin, a reduction in nasolabial hypertrophy, and an improvement in the contour of the mandibular and mentocervical lines.

SLEB is an ultrasound indicator associated with collagen degradation and dermal inflammation, as evidenced by decreased echogenicity [3,4]. Increased echogenicity suggests collagen remodeling and an anti-aging effect following adipostructuring. Histopathological studies have shown that the technique is truly effective in stimulating collagen fibers, effectively improving skin structure [5,6]. Ultrasound imaging has demonstrated a reduction in aging and an improvement in the location and structure of adipose tissue.

For its part, ultrasound transforms the qualitative perception of changes into quantitative, statistically analyzable, and reproducible data, demonstrating clinical efficacy before and after the procedure, as well as revealing specific parameters in skin thickness, echogenicity, and echostructure at each tissue interface. This allows changes in the arrangement pattern to be demonstrated. Therefore, it is undeniable that the technique is aligned with cellular evidence studies, making it an effective modality for facial rejuvenation [6,7]. We can infer that the procedure, in addition to being safe [8], can be recommended as a useful tool in the skin aging process. With this knowledge, facial adipostructuring, as a new clinical approach, demonstrates its efficacy in treating the stigmatizing signs that appear with age, including increased dermal thickness.

## Conclusions

Understanding the biomechanical and anatomical principles of the face allows for more precise and personalized results, minimizing risks and maximizing naturalness. This enables us to affirm that facial adipostructuring represents a modern and effective approach that allows for metabolic induction in facial fat tissue, accompanied by fibrillar stimulation, promoting the natural repositioning of tissues. It is an approach that improves the signs of aging, preparing the structures for other treatments. Ultrasound not only acts as an intraoperative map but also as a scientific validation tool that allows for the quantification and statistical demonstration of the benefits of facial rejuvenation, elevating fat grafting to a rigorously evidence-based intervention. Furthermore, one of its primary objectives is diagnosis, making it a fundamental tool for managing complications, the most frequent being edema and hematomas. It also provides specialists in this field with the assurance that they are not facing other types of complications. The facial adipostructuring, as a clinical approach based on the philosophy of intelligent rejuvenation, not only improves aesthetic appearance but also positively influences patients' self-esteem and emotional well-being. This is despite the existence of biological parameters associated with masculinity and femininity.
